# Sex Differences in Psychiatric Comorbidity and Plasma Biomarkers for Cocaine Addiction in Abstinent Cocaine-Addicted Subjects in Outpatient Settings

**DOI:** 10.3389/fpsyt.2015.00017

**Published:** 2015-02-16

**Authors:** María Pedraz, Pedro Araos, Nuria García-Marchena, Antonia Serrano, Pablo Romero-Sanchiz, Juan Suárez, Estela Castilla-Ortega, Fermín Mayoral-Cleries, Juan Jesús Ruiz, Antoni Pastor, Vicente Barrios, Julie A. Chowen, Jesús Argente, Marta Torrens, Rafael de la Torre, Fernando Rodríguez De Fonseca, Francisco Javier Pavón

**Affiliations:** ^1^Unidad Gestión Clínica de Salud Mental, Instituto de Investigación Biomédica de Málaga (IBIMA), Hospital Regional Universitario de Málaga, Universidad de Málaga, Málaga, Spain; ^2^Centro Provincial de Drogodependencia, Diputación de Málaga, Málaga, Spain; ^3^Institut Hospital del Mar d’Investigacions Mèdiques (IMIM), Barcelona, Spain; ^4^Facultat de Medicina, Universitat Autonoma de Barcelona, Barcelona, Spain; ^5^Department of Pediatrics and Pediatric Endocrinology, Hospital Infantil Universitario Niño Jesús, Universidad Autónoma de Madrid, Madrid, Spain; ^6^Centro de Investigación Biomédica en Red de la Fisiopatología de la Obesidad y Nutrición (CIBEROBN), Instituto de Salud Carlos III, Madrid, Spain; ^7^Institut de Neuropsiquiatria i Addiccions (INAD) del Parc de Salut MAR, Barcelona, Spain; ^8^Facultat de Ciencies de la Salut i de la Vida, Universitat Pompeu Fabra (CEXS-UPF), Barcelona, Spain

**Keywords:** cocaine use disorders, psychiatric comorbidity, cytokine, endocannabinoid, sex, outpatient, biomarker, abstinence

## Abstract

There are sex differences in the progression of drug addiction, relapse, and response to therapies. Because biological factors participate in these differences, they should be considered when using biomarkers for addiction. In the current study, we evaluated the sex differences in psychiatric comorbidity and the concentrations of plasma mediators that have been reported to be affected by cocaine. Fifty-five abstinent cocaine-addicted subjects diagnosed with lifetime cocaine use disorders (40 men and 15 women) and 73 healthy controls (48 men and 25 women) were clinically assessed with the diagnostic interview “Psychiatric Research Interview for Substance and Mental Disorders.” Plasma concentrations of chemokines, cytokines, *N*-acyl-ethanolamines, and 2-acyl-glycerols were analyzed according to history of cocaine addiction and sex, controlling for covariates age and body mass index (BMI). Relationships between these concentrations and variables related to cocaine addiction were also analyzed in addicted subjects. The results showed that the concentrations of chemokine (C-C motif) ligand 2/monocyte chemotactic protein-1 (CCL2/MCP-1) and chemokine (C-X-C motif) ligand 12/stromal cell-derived factor-1 (CXCL12/SDF-1) were only affected by history of cocaine addiction. The plasma concentrations of interleukin 1-beta (IL-1β), IL-6, IL-10, and tumor necrosis factor-alpha (TNFα) were affected by history of cocaine addiction and sex. In fact, whereas cytokine concentrations were higher in control women relative to men, these concentrations were reduced in cocaine-addicted women without changes in addicted men. Regarding fatty acid derivatives, history of cocaine addiction had a main effect on the concentration of each acyl derivative, whereas *N*-acyl-ethanolamines were increased overall in the cocaine group, 2-acyl-glycerols were decreased. Interestingly, *N*-palmitoleoyl-ethanolamine (POEA) was only increased in cocaine-addicted women. The covariate BMI had a significant effect on POEA and *N*-arachidonoyl-ethanolamine concentrations. Regarding psychiatric comorbidity in the cocaine group, women had lower incidence rates of comorbid substance use disorders than did men. For example, alcohol use disorders were found in 80% of men and 40% of women. In contrast, the addicted women had increased prevalences of comorbid psychiatric disorders (i.e., mood, anxiety, and psychosis disorders). Additionally, cocaine-addicted subjects showed a relationship between the concentrations of *N*-stearoyl-ethanolamine and 2-linoleoyl-glycerol and diagnosis of psychiatric comorbidity. These results demonstrate the existence of a sex influence on plasma biomarkers for cocaine addiction and on the presence of comorbid psychopathologies for clinical purposes.

## Introduction

Over the last 10 years, cocaine has established itself as the most commonly used illicit stimulant drug in Europe, although most users are found in a small number of high-prevalence countries. Cocaine use is particularly high in Spain, with a lifetime prevalence of 10.2% in the general population, and it represents a significant public health concern (1).

There are several factors that influence the acquisition, maintenance, and progression to addiction, such as social context, age, genetic characteristics, and sex (2). In this respect, sex differences have been found in cocaine use and addiction, including cocaine use initiation, progression to abuse and dependence, relapse following abstinence, and responsiveness to treatment (3–5).

Epidemiological data suggest that women have a rapid escalation in drug use and progress more quickly to cocaine addiction compared with men (6). Women are more sensitive to social stressors, and abstinent cocaine-addicted women report higher levels of craving in response to cocaine-related cues (7, 8). Sex also influences treatments and relapses because women report shorter abstinence periods and higher relapse rates after stressful or depressive events (9). All of these observations parallel preclinical animal models using rodents showing that females are more vulnerable to the abuse-related effects of cocaine than males (10).

Despite evidence that women are more vulnerable than men to cocaine addiction, the rates of cocaine use are currently higher in men than in women, and the proportion of cocaine users seeking treatment in outpatient cocaine programs is approximately five men to every woman in Europe (1). Considering that cocaine addiction is commonly associated with altered executive functions, impaired emotional processing capacity, and elevated incidence of comorbid mental disorders (11, 12), sex is a primary factor underlying these behavioral complications. In fact, sex differences in psychopathologies and substance use disorders have been linked to the activity of hormones (i.e., gonadal steroid hormones), menstrual cycle, HPA axis reactivity, and neurobiological factors (13–15).

Recently, the search for biomarkers for psychiatric disorders and addiction has generated a number of putative biomarkers that includes circulating mediators with neuromodulatory functions (16–18). Among these molecules, inflammatory proteins and fatty acid derivatives have been reported to be altered in abstinent cocaine-addicted subjects (19, 20). Moreover, changes in plasma cytokine and chemokine concentrations have been shown to be related to the pathological cocaine use and cocaine symptom severity (20), whereas changes in endocannabinoids and congeners are related to cocaine use disorders and psychiatric comorbidity (19). However, the influence of sex was not directly studied in these reports.

Because previous studies have reported sex differences in all phases related to the progression to cocaine addiction and because gonadal hormones can affect other signaling systems sensitive to cocaine addiction, those molecules identified as putative biomarkers for cocaine addiction and common psychiatric comorbidity may be influenced by sex.

The primary purpose of the present observational study was to examine the plasma concentrations of chemokines, cytokines, and fatty acid derivatives in a cohort of abstinent cocaine-addicted subjects on an outpatient basis according to their sex. Additionally, the prevalences of comorbidity of other substance and mental disorders were evaluated.

## Materials and Methods

### Subjects and recruitment

All participants were white Caucasians grouped into abstinent cocaine users and healthy controls. Fifty-nine cocaine users (17 women and 42 men) were initially enrolled from outpatient treatment programs for cocaine addiction in the province of Málaga (Spain) for a 24-month period (2011–2013). Seventy-six healthy individuals (25 women and 51 men) were recruited from a multidisciplinary staff working at the Hospital Regional Universitario de Málaga.

Cocaine users had to meet eligibility criteria based on inclusion and exclusion criteria. Inclusion criteria were as follows: ≥18–65 years of age, intranasal cocaine use, diagnosis of lifetime cocaine use disorders, and abstinence from cocaine for at least 2 weeks before testing (urine and plasma analyses). Exclusion criteria were as follows: personal history of chronic diseases (e.g., cardiovascular, respiratory, renal, hepatic, neurological, or endocrine diseases), personal history of cancer, infectious diseases, incapacitating cognitive alterations, and pregnancy.

Controls were matched with the cocaine group for sex ratio, age, and body mass index (BMI). In addition to the mentioned exclusion criteria for abstinent cocaine users, controls were excluded if they had a personal history of drug abuse or lifetime psychiatric disorders. All women were recruited without considering their menstrual cycle.

Finally, 55 abstinent cocaine-addicted subjects (15 women and 40 men) and 63 controls (25 women and 48 men) met the eligibility criteria and completed the study.

All cocaine-addicted subjects were under current treatment interventions, including pharmacological and behavioral approaches. Regarding the pharmacological interventions, 20 participants (12 men and 8 women) were treated with anxiolytics (*n* = 9), antipsychotics (*n* = 2), antidepressants (*n* = 8), and disulfiram (*n* = 1).

### Clinical assessments

Cocaine users were evaluated according to “Diagnostic and Statistical Manual of Mental Disorders-4th Edition-Text Revision” (DSM-IV-TR) criteria, using the Spanish version of the “Psychiatric Research Interview for Substance and Mental Disorders” (PRISM) (21, 22). Controls were evaluated by PRISM (for substance screening and abuse and dependence) and the Spanish version of the “Composite International Diagnostic Interview” (CIDI) to detect psychiatric disorders (23). All interviews were performed by experienced psychologists who had received both PRISM and CIDI training. Two cocaine users did not meet the criteria for cocaine use disorders and three controls were diagnosed with lifetime mental disorders (major depression and anxiety). They were consequently excluded from the study.

#### Psychiatric research interview for substance and mental disorders (PRISM)

The PRISM is a semistructured interview to diagnose psychiatric disorders among substance users (22, 24, 25). Diagnoses were made using two time-frames: “current” (criteria were met within the past year) and “past” (criteria were met before the previous 12 months). Lifetime prevalence was used to present the frequency of substance use disorders, non-substance use disorders, and psychiatric comorbidity. The cocaine symptom severity was assessed combining the DSM-IV-TR criteria for cocaine use disorders: 7 dependence criteria (for a diagnosis of dependence, 3 or more co-occurring symptoms in a 12-month period are required) and 4 abuse criteria (1 symptom is necessary for a diagnosis of abuse) (19, 20).

### Laboratory methods for human samples

#### Collection and analysis of plasma samples

Blood samples were obtained in the morning (09:00–11:00 a.m.) after fasting for 8–12 h (previous to the psychiatric interviews). Venous blood was collected into 10 mL K_2_-EDTA tubes (BD, Franklin Lakes, NJ, USA) and processed to obtain plasma. Blood samples were centrifuged at 2,200 × *g* for 15 min (4°C), and plasma was analyzed for HIV and hepatitis types B and C.

##### Analysis for HIV and hepatitis types B and C

Plasmas samples were individually assayed by three rapid tests for detecting HIV (Retroscreen HIV, QualPro Diagnostics-Tulip Group Ltd., Goa, India), hepatitis B (HBsAg Test, Toyo Diagnostics-Turklab Inc., Izmir, Turkey), and hepatitis C (Flaviscreen HCV, QualPro Diagnostics-Tulip Group Ltd., Goa, India). No samples that tested positive were detected. Plasma samples were stored at −80°C until further analyses.

##### Analysis for the cocaine metabolite benzoylecgonine

Plasma analyses for cocaine metabolites (Benzoylecgonine Specific Direct ELISA Kit Immunalysis, Pomona, CA, USA) were performed to confirm cocaine abstinence. Two cocaine users who tested negative for drugs of abuse in urine analyses at the outpatient treatment centers for cocaine addiction were positive for benzoylecgonine in plasma, and they were excluded from this study.

#### Multiplex immunoassay analysis

A Bio-Plex Suspension Array System 200 (Bio-Rad Laboratories, Hercules, CA, USA) was used to quantify the plasma concentrations of inflammatory cytokines and chemokines following the manufacturer’s instructions as previously reported (20). Human protein panels were used to simultaneously detect the following analytes: tumor necrosis factor-alpha (TNFα); interleukin-1 beta (IL-1β); interleukin-6 (IL-6); interleukin-10 (IL-10); chemokine (C-X_3_-C motif) ligand 1 [CX3CL1], commonly referred to as fractalkine; chemokine (C-C motif) ligand 2 [CCL2], also referred to as monocyte chemotactic protein-1 (MCP-1); and chemokine (C-X-C motif) ligand 12 [CXCL12], also referred to stromal cell-derived factor-1 (SDF-1). Raw data (mean fluorescence intensity) were analyzed using the Bio-Plex Manager Software 4.1 (Bio-Rad Laboratories, Hercules, CA, USA). Data of plasma concentrations were in pg of protein per mL of plasma.

#### Quantification of fatty acid derivatives

The following fatty acid derivatives and their respective deuterated forms were used for quantification: *N*-stearoyl-ethanolamine (SEA), *N*-palmitoyl-ethanolamine (PEA) and PEA-d_4_, *N*-oleoyl-ethanolamine (OEA) and OEA-d_4_, *N*-palmitoleoyl-ethanolamine (POEA), *N-*arachidonoyl-ethanolamine (AEA) and AEA-d_4_, *N*-linoleoyl-ethanolamine (LEA) and LEA-d_4_, 2-arachidonoyl-glycerol (2-AG) and 2-AG-d_5_, and 2-linoleoyl-glycerol (2-LG). PEA-d_4_ and OEA-d_4_ were used for the quantification of POEA and SEA, respectively, because their deuterated forms were not commercially available. All reagents were obtained from Cayman Chemical (Ann Arbor, MI, USA).

Sample extraction and chromatographic separation were performed in a liquid chromatography-tandem mass spectrometry system (Agilent Technologies, Wilmington, DE, USA) as previously reported (19, 26). Data of plasma concentrations were in ng of acyl derivative per mL of plasma.

### Ethics statement

Written informed consent was obtained from each subject after they had received a complete description of the present study and had been given the chance to discuss any questions or issues. The study and protocols for recruitment were approved by the Ethics Committee of the Hospital Regional Universitario de Málaga (07/19/2009 PND049/2009 and PI0228-2013; CEI Provincial de Málaga) and were therefore conducted in accordance with the Declaration of Helsinki (seventh revision in 2013, Fortaleza, Brazil).

### Statistical analyses

All data for graphs and tables are expressed as number and percentage of subjects [*n* (%)] or the mean and standard deviation [mean (SD)]. Samples were grouped by sex and the significance of differences was assessed by Fisher’s exact test or Student’s *t*-test. The statistical analyses of plasma concentrations were performed using two-way analysis of covariance (ANCOVA) [factors: history of cocaine addiction (cocaine/control) and sex (men/women); covariates: age and BMI]. Multiple comparisons were performed with unadjusted (observed) or adjusted (estimated marginal) according to the effects of covariates. Plasma concentrations in subjects with a history of cocaine addiction were analyzed by univariate general linear models to evaluate relationships with sex, age, BMI, and group-specific variables (cocaine symptom severity, diagnosis of comorbid psychiatric disorders, and length of cocaine abstinence). A *p*-value less than 0.05 was considered statistically significant. Statistical analyses were performed using the GraphPad Prism version 5.04 software (GraphPad Software, San Diego, CA, USA) and IBM SPSS Statistical version 22 software (IBM, Armonk, NY, USA).

## Results

### Social and demographic characteristics

A total of 128 subjects met the eligibility criteria for this study and were grouped into the cocaine (*n* = 55) and control (*n* = 73) groups. Both groups were divided into men and women. A description of the sample is presented in Table 1.

**Table 1:** **Baseline socio-demographic variables in abstinent cocaine-addicted and control subjects grouped by sex**.

Variable		Cocaine group		Control group	
		Men	Women	Men	Women
Sex [*n* (%)]		40 (72.7)	15 (27.3)	48 (65.8)	25 (34.2)
Age (≥18) [Mean (SD)]		37.1 (6.7)	42.8 (6.2)*	38.6 (9.8)	42.6 (8.4)
Body mass [Mean (SD)]	Body mass index	26.2 (4.3)	25.4 (5.9)	25.4 (5.9)	23.9 (4.7)
Current marital status [*n* (%)]	Never married	12 (30.0)	4 (26.7)	23 (47.9)	10 (40.0)
	Married/cohabiting	19 (47.5)	7 (46.7)	22 (45.8)	10 (40.0)
	Divorced/separated	9 (22.5)	3 (20.0.)	3 (6.3)	4 (16.0)
	Widowed	0 (0.0)	1 (6.7)	0 (0.0)	1 (4.0)
Living together last year [*n* (%)]	Friends, squatters	1 (2.5)	1 (6.7)	3 (6.3)	0 (0.0)
	Parents	14 (35.0)	5 (33.3)	9 (18.8)	7 (28.0)
	Couple	20 (50.0)	7 (46.7)	25 (52.1)	12 (48.0)
	Alone	4 (10.0)	1 (6.7)	10 (20.8)	6 (24.0)
	Others	1 (2.5)	1 (6.7)	1 (2.1)	0 (0.0)
Educational level [*n* (%)]	≤ Primary level	24 (60.0)	8 (53.3)	3 (6.3)	0 (0.0)
	≥ Secondary level	16 (40.0)	7 (46.7)	45 (93.8)	25 (100.0)
Work status [*n* (%)]	Employed	16 (40.0)	4 (26.7)	42 (87.5)	23 (92.0)
	Unemployed	22 (55.0)	10 (66)	5 (10.4)	2 (8.0)
	Retired/disabled	2 (5.0)	1 (6.7)	0 (0.0)	0 (0.0)
	Student	0 (0.0)	0 (0.0)	1 (2.1)	0 (0.0)
Use of psychological resources [*n* (%)]	No	28 (70.0)	6 (40.0)	44 (91.7)	13 (52.0)
	Yes	12 (30.0)	9 (60.0)	4 (8.3)	12 (48.0)

*p < 0.05 denotes significant differences between cocaine-addicted men and women.

Men seeking treatment for cocaine addiction were more common than women, at a ratio of one woman to eight men in the centers for cocaine addiction where the recruitment was conducted (data not shown). During a 24-month period (January 2011–December 2012), 20 women were contacted to participate in this study; 17 accepted and were diagnosed with lifetime cocaine use disorders. Finally, 15 cocaine-addicted women completed the study. Whereas the abstinent cocaine-addicted men had a mean age of 37 years, the cocaine-addicted women were older (mean: 43 years; *p* < 0.01). We observed no differences between the sexes in other socio-demographic variables in the cocaine group. Considering both sexes, the addicted subjects were married/cohabiting (47%), lived in couple (49%), had a low educational level (42% with secondary level or more), and were unemployed (58%). In contrast, the control subjects had a higher educational level (96% with secondary level or more) and employment rate (89%).

Interestingly, the cocaine-addicted subjects displayed a higher incidence in the use of psychological counseling, excluding treatments of severe/serious mental disorders, with 30% in men and 60% in women. These percentages were reduced in the control group to 8% in men and 48% in women.

### Comorbid mental and substance use disorders in abstinent cocaine-addicted subjects grouped by sex

#### Comorbid substance use disorders and cocaine use-related variables

As shown in Table 2, the cocaine group had an elevated prevalence of comorbid substance use disorders in addition to cocaine use disorders, primarily alcohol (69%) and cannabis (20%) use disorders. We observed sex differences in the prevalences of these other substance use disorders because they were more common in men than in women: alcohol use disorders were diagnosed in 80% of men and 40% of women (*p* < 0.01), and cannabis use disorders were diagnosed in 25% of men and 7% of women.

**Table 2:** **Cocaine use-related variables in abstinent cocaine-addicted subjects grouped by sex**.

Variable		Cocaine group		*p*-Value
		Men (*n* = 40)	Women (*n* = 15)	
Lifetime substance use disorders [*n* (%)]	Cocaine use disorders	40 (100.0)	15 (100.0)	ns
	Alcohol use disorders	32 (80.0)	6 (40.0)	**0.008**
	Cannabis use disorders	10 (25.0)	1 (6.7)	ns
	Other substance use disorders	9 (22.5)	2 (13.3)	ns
Lifetime cocaine use disorders [*n* (%)]	Cocaine abuse	35 (87.5)	14 (93.3)	ns
	Cocaine dependence	36 (90.0)	13 (86.7)	ns
	Cocaine abuse and dependence	31 (77.5)	12 (80.0)	ns
Cocaine abstinence [Mean (SD)]	Days	184.2 (323.2)	163.7 (145.2)	ns
Cocaine use [Mean (SD)]	Years	8.0 (6.8)	9.6 (6.5)	ns
DSM-IV criteria for cocaine use disorders [Mean (SD)]	Counts	8.1 (2.5)	7.9 (1.9)	ns

*ns, non-significant*.

*Bold indicates statistically significant *p*-values*.

Focusing on the variables related to cocaine use, we did not observe sex differences in the prevalences of abuse and dependence, length of cocaine abstinence, duration of cocaine use, or cocaine symptom severity. Therefore, the average cocaine-addicted subject, including men and women, displayed cocaine abstinence for 178.6 (281.7) days [mode: 120 days (range: 730)], a cumulative cocaine use of 8.2 (6.6) years [mode: 4 years (range: 31)] and 8.1 (2.5) DSM-IV criteria for cocaine use disorders.

#### Comorbid mental disorders

Regarding the common psychiatric disorders assessed with the PRISM, we found high prevalences of comorbid psychopathologies (60%): mood (38%), anxiety (22%), psychosis (20%), and personality (35%) disorders.

According to sex, cocaine-addicted women showed higher prevalences of mood, anxiety (*p* < 0.05), and psychosis disorders compared with cocaine-addicted men (Table 3). Though some women were diagnosed with both primary and cocaine-induced disorders for mood and anxiety disorders, no men were diagnosed with primary and cocaine-induced disorders (the two together). However, these psychiatric prevalences were not significantly different between men and women, although there was a limitation in the sample size considered.

**Table 3:** **Psychiatric comorbidity in abstinent cocaine-addicted subjects grouped by sex**.

Variable		Cocaine group		*p*-value
		Men (*n* = 40)	Women (*n* = 15)	
Lifetime psychiatric disorders^a^ [*n* (%)]	No	18 (45.0)	4 (26.7)	ns
	Mood disorders	13 (32.5)	8 (53.3)	ns
	Anxiety disorders	5 (12.5)	7 (46.7)	**0.011**
	Psychosis disorders	6 (15.0)	5 (33.3)	ns
	Eating disorders	0 (0.0)	0 (0.0)	–
	Personality disorders	14 (35.0)	5 (33.3)	ns
Mood disorders [*n* (%)]	Primary	6 (15.0)	2 (13.3)	ns
	Cocaine-induced	7 (17.5)	5 (33.3)	
	Primary and cocaine-induced	0 (0.0)	1 (6.7)	
Anxiety disorders [*n* (%)]	Primary	2 (5.0)	3 (20.0)	ns
	Cocaine-induced	3 (7.5)	2 (13.3)	
	Primary and cocaine-induced	0 (0.0)	2 (6.7)	
Psychosis disorders [*n* (%)]	Primary	0 (0.0)	0 (0.0)	ns
	Cocaine-induced	6 (15.0)	5 (33.3)	
	Primary and cocaine-induced	0 (0.0)	0 (0.0)	
Personality disorders [*n* (%)]	Borderline	6 (15.0)	3 (20.0)	ns
	Antisocial	6 (15.0)	1 (6.7)	
	Borderline and antisocial	2 (5.0)	1 (6.7)	

*^a^Psychiatric disorders diagnosed with PRISM*.

*ns, non-significant*.

*Bold indicates statistically significant *p*-values*.

Regarding personality disorders, we observed no differences between the sexes.

### Plasma concentrations of chemokines according to history of cocaine addiction and sex

**Figure 1:**
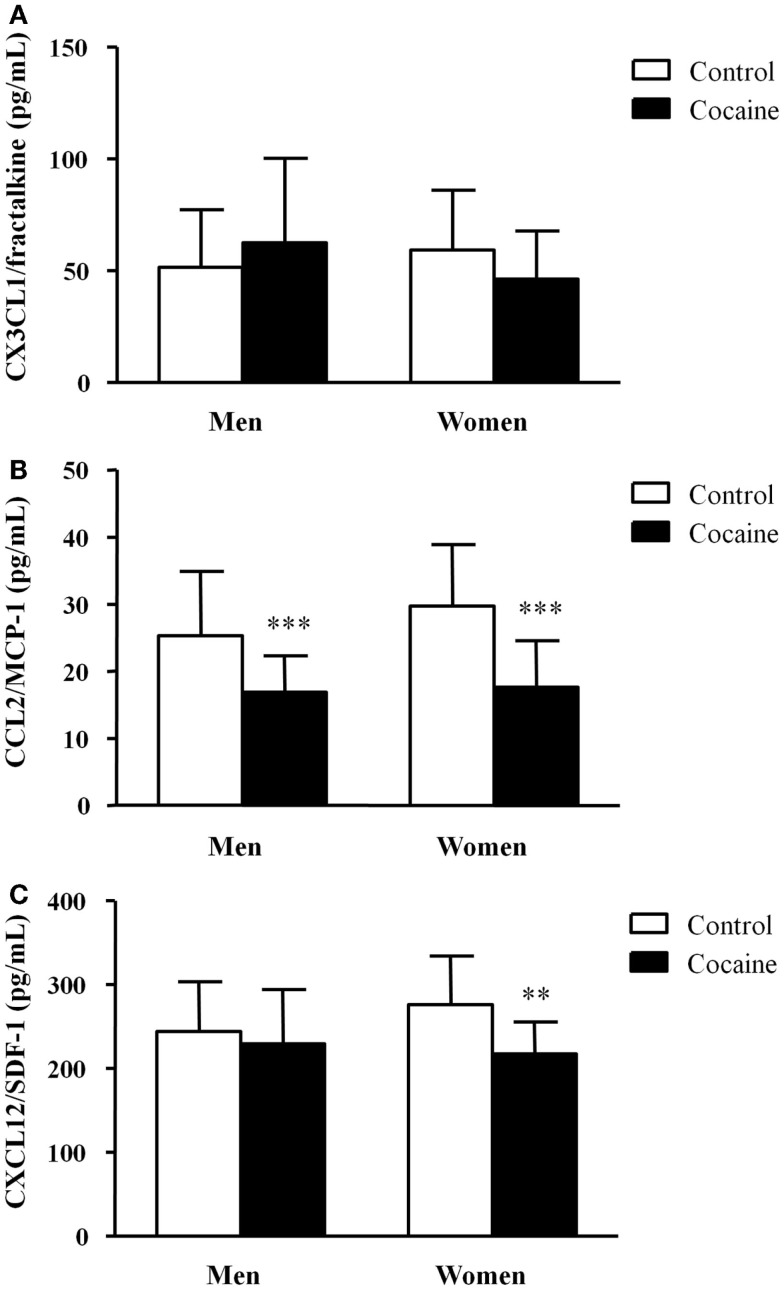
**Plasma concentrations of chemokines in abstinent cocaine-addicted and control subjects grouped by sex**. **(A)** CX3CL1/fractalkine (pg/mL); **(B)** CCL2/MCP-1 (pg/mL); and **(C)** CXCL12/SDF-1 (pg/mL). The bars represent means ± SD. The data were analyzed by two-way ANCOVA and multiple comparison tests. (**) *p* < 0.01 and (***) *p* < 0.001 denote significant differences compared with the respective control men or control women.

The mean plasma concentrations of CX3CL1/fractalkine, CCL2/MCP-1, and CXCL12/SDF-1 in the cocaine and control groups are shown in Figure 1. Two-way ANCOVAs were performed to analyze the chemokine concentrations. Covariates age and BMI were included in the analyses but there was not a significant effect of the covariates on either chemokine concentrations.

We did not observe main effects of history of cocaine addiction and sex on plasma CX3CL1 concentrations, but there was a significant interaction effect (*F*_1,122_ = 4.60, *p* = 0.034) (Figure 1A). However, the plasma concentrations of CCL2 were significantly affected by history of cocaine addiction (*F*_1,122_ = 42.03, *p* < 0.001) (Figure 1B). The multiple comparisons showed that cocaine-addicted men and women had significantly decreased CCL2 concentrations (***p < 0.001) compared with their respective controls. Similarly, history of cocaine addiction (*F*_1,122_ = 7.72, *p* = 0.006) had a significant main effect on CXCL12 concentrations (Figure 1C). In this case, we detected a significant decrease in cocaine-addicted women (**p < 0.01) compared with control women, but this difference was not found in men.

Therefore, sex had no significant primary effect on chemokine concentrations.

### Plasma concentrations of cytokines according to history of cocaine addiction and sex

**Figure 2:**
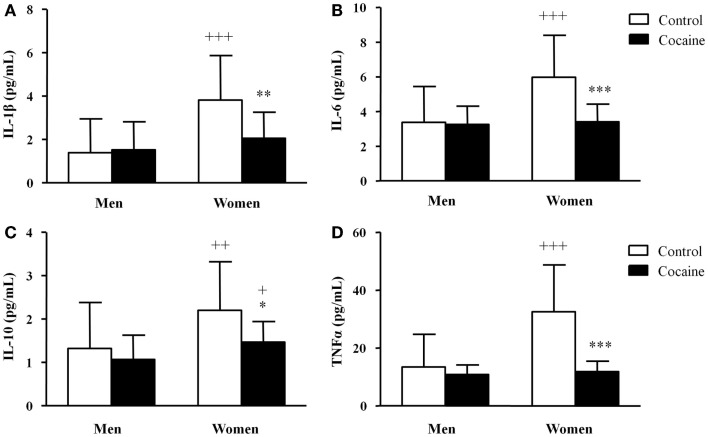
**Plasma concentrations of cytokines in abstinent cocaine-addicted and control subjects grouped by sex**. **(A)** IL-1β (pg/mL); **(B)** IL-6 (pg/mL); **(C)** IL-10 (pg/mL); and **(D)** TNFα (pg/mL). The bars represent means ± SD. The data were analyzed by two-way ANCOVA and multiple comparison tests. (*) *p* < 0.05, (**) *p* < 0.01, and (***) *p* < 0.001 denote significant differences compared with control women. (+) *p* < 0.05, (++) *p* < 0.01, and (+ + +) *p* < 0.001 denote significant differences compared with the respective control men or cocaine-addicted men.

The plasma concentrations of IL-1β, IL-6, IL-10, and TNFα in the cocaine and control groups are illustrated in Figure 2. Again, there was not a significant relationship between the covariates and cytokine concentrations.

As shown in Figure 2A, the IL-1β concentrations were significantly affected by history of cocaine addiction (*F*_1,122_ = 5.73, *p* = 0.018) and by sex (*F*_1,122_ = 20.41, *p* < 0.001). An interaction between history of cocaine addiction and sex was also detected (*F*_1,122_ = 8.36, *p* = 0.005). The paired comparisons revealed that control women had significantly increased IL-1β concentrations (+ + + *p* < 0.001) compared with control men (3.81 ± 2.06 and 1.40 ± 1.55 pg/mL, respectively). However, cocaine-addicted women had a significant decrease in the IL-1β concentrations (**p < 0.01) relative to control women but exhibited no differences compared with cocaine-addicted men.

The IL-6 concentrations (Figure 2B) were also significantly affected by history of cocaine addiction (*F*_1,122_ = 13.46, *p* < 0.001) and sex (*F*_1,122_ = 11.05, *p* = 0.001). Additionally, there was a significant interaction between the factors (*F*_1,122_ = 13.35, *p* < 0.001). Thus, although control women had higher IL-6 concentrations (+ + + *p* < 0.001) than did control men (5.98 ± 2.41 and 3.37 ± 2.08 pg/mL, respectively), the IL-6 concentrations in cocaine-addicted women were significantly lower (***p < 0.001) than in control women.

In relation to IL-10 (Figure 2C), history of cocaine addiction (*F*_1,122_ = 8.69, *p* = 0.004) and sex (*F*_1,122_ = 11.14, *p* = 0.001) had a significant primary effect on the IL-10 concentrations. There was an interaction between the factors (*F*_1,122_ = 25.63, *p* < 0.001). Considering the *post hoc* comparisons, control women had increased IL-10 concentrations (+ + *p* < 0.01) relative to control men (2.20 ± 1.12 and 1.32 ± 1.06 pg/mL, respectively). In addition, cocaine-addicted women exhibited a significant decrease in IL-1β concentrations (*p < 0.05) compared with control women, but cocaine-addicted women had higher concentrations (+ *p* < 0.05) than did cocaine-addicted men.

Finally, the statistical analysis of TNFα concentrations (Figure 2D) revealed a significant main effect of history of cocaine addiction (*F*_1,122_ = 34.98, *p* < 0.001) and sex (*F*_1,122_ = 20.90, *p* < 0.001) after adjusting for covariates. There was an interaction between history of cocaine addiction and sex (*F*_1,122_ = 22.46, *p* < 0.001). Once again, control women had higher TNFα concentrations (+ + + *p* < 0.001) than did control men (32.55 ± 16.21 and 13.47 ± 11.28 pg/mL, respectively). Cocaine-addicted women showed a significant decrease in TNFα concentrations (***p < 0.001) compared with control women, but no differences compared with cocaine-addicted men.

Therefore, we observed higher cytokine concentrations in women than in men. Moreover, a specific and dramatic decrease in cytokine concentrations was observed in cocaine-addicted women but not in cocaine-addicted men.

### Plasma concentrations of fatty acid derivatives according to history of cocaine addiction and sex

**Figure 3:**
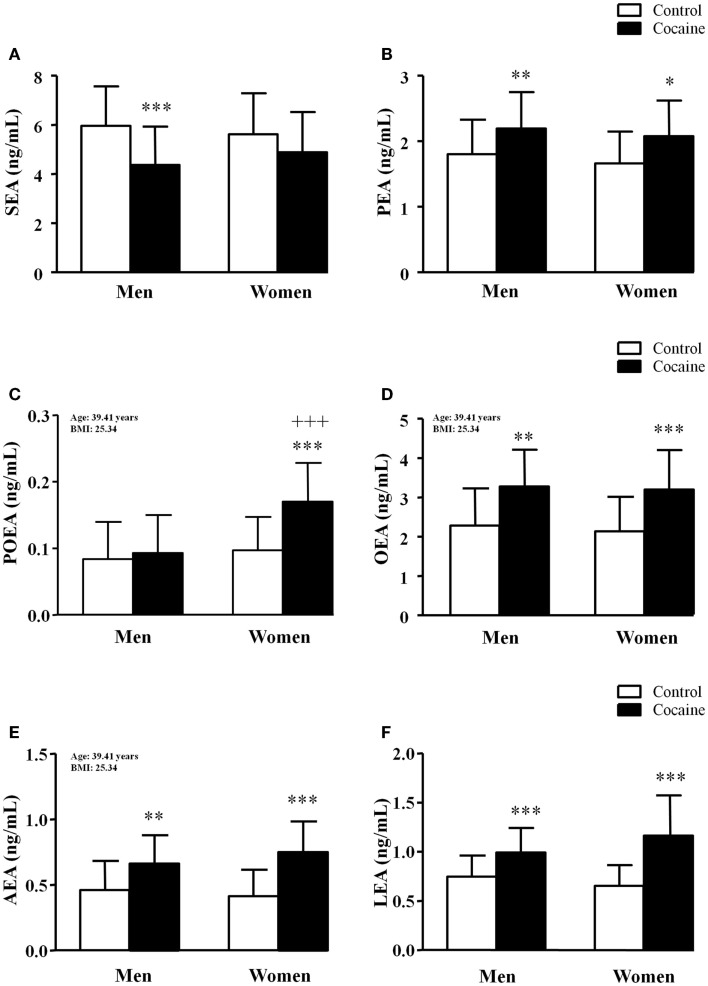
**Plasma concentrations of *N*-acyl-ethanolamines in abstinent cocaine-addicted and control subjects grouped by sex**. **(A)** SEA (ng/mL); **(B)** PEA (ng/mL); **(C)** POEA (ng/mL); **(D)** OEA (ng/mL); **(E)** AEA (ng/mL); and **(F)** LEA (ng/mL). The bars represent means ± SD. The data were analyzed by two-way ANCOVA and multiple comparison tests. (*) *p* < 0.05, (**) *p* < 0.01, and (***) *p* < 0.001 denote significant differences compared with the respective control men or control women. (+++) *p* < 0.001 denotes significant differences compared with cocaine-addicted men. POEA, OEA, and AEA concentrations are adjusted means with the following values of covariates: age = 39.41 years and BMI = 25.34.

**Figure 4:**
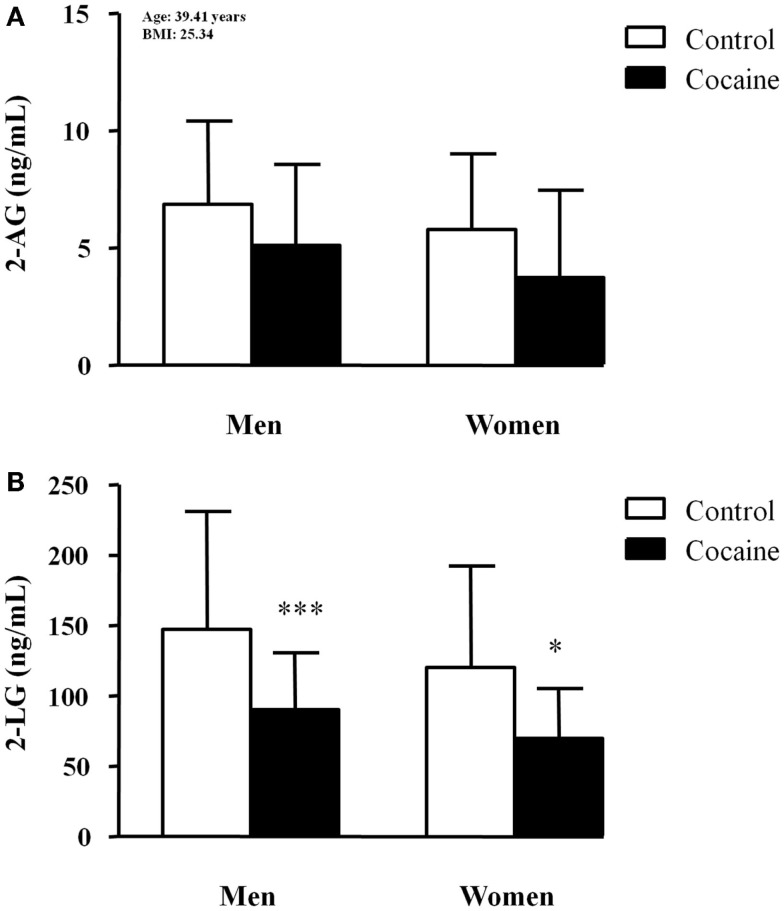
**Plasma concentrations of 2-acyl-glycerols in abstinent cocaine-addicted and control subjects grouped by sex**. **(A)** 2-AG (ng/mL); and **(B)** 2-LG (ng/mL). The bars represent means ± SD. The data were analyzed by two-way ANCOVA and multiple comparison tests. (*) *p* < 0.05 and (***) *p* < 0.001 denote significant differences compared with the respective control men or control women. 2-AG concentrations are adjusted means with the following values of covariates: age = 39.41 years and BMI = 25.34.

The plasma concentrations of fatty acid derivatives were grouped into the following categories: (i) saturated (SEA and PEA), monounsaturated (POEA and OEA), and polyunsaturated (AEA and LEA) *N*-acyl-ethanolamines (Figure 3) and (ii) 2-acyl-glycerols (2-AG and 2-LG) (Figure 4). Significant relationships between the covariates (age and BMI) and these fatty acid derivative concentrations were detected by the statistical analysis.

#### *N*-acyl-ethanolamines

History of cocaine addiction (*F*_1,122_ = 11.09, *p* = 0.001) had a significant main effect on plasma SEA concentrations, but sex had no effect (Figure 3A). There was not a significant effect of the covariates on SEA concentrations, and the *post hoc* tests using unadjusted means only indicated a significant decrease in the cocaine-addicted men (***p < 0.001) compared with the control men. Regarding the other saturated lipid derivative (Figure 3B), plasma PEA concentrations were also significantly affected by history of cocaine addiction (*F*_1,122_ = 11.04, *p* = 0.001) without effect of the covariates. However, cocaine-addicted subjects showed significantly higher plasma PEA concentrations than did controls in both men (*p < 0.01) and women (**p < 0.01).

A two-way ANCOVA revealed a significant primary effect of history of cocaine addiction (*F*_1,122_ = 13.79, *p* < 0.001) and sex (*F*_1,122_ = 16.07, *p* < 0.001) on plasma POEA concentrations (Figure 3C). Additionally, there was a significant interaction between history of cocaine addiction and sex (*F*_1,122_ = 8.45, *p* = 0.004). In this case, there was a significant relationship between BMI and POEA concentrations (*F*_1,122_ = 5.11, *p* = 0.026), and BMI explained 4.7% of the variance. Consequently, all pairwise comparisons were performed using estimated marginal means with fixed values of covariates (age = 39.41 and BMI = 25.34). The confidence interval (CI) adjustments for the Bonferroni test indicated the following estimated marginal means of POEA concentrations and standard errors: 0.084 ± 0.008 ng/mL (0.068–0.100, 95% CI) in control men, 0.097 ± 0.010 ng/mL (0.077–0.11, 95% CI) in control women, 0.093 ± 0.009 ng/mL (0.076–0.110, 95% CI) in cocaine-addicted men and 0.170 ± 0.015 ng/mL (0.139–0.200, 95% CI) in cocaine-addicted women. Multiple comparisons with adjusted means indicated that the main effect of history of cocaine addiction was exclusively observed in cocaine-addicted women. A marked increase in plasma POEA concentrations was observed in cocaine-addicted women (***p < 0.001) relative to control women but also in comparison with cocaine-addicted men (+ + + *p* < 0.001). There were no changes in POEA concentrations in men.

Regarding the other monounsaturated lipid derivative (Figure 3D), plasma OEA concentrations were only affected by cocaine use (*F*_1,122_ = 30.73, *p* < 0.001). There was a significant relationship between age and OEA concentrations (*F*_1,122_ = 6.84, *p* = 0.010), and the covariate explained 6.2% of the variance. The adjusted means (age = 39.41 and BMI = 25.34) of OEA concentrations and standard errors of the means were as follows: 2.263 ± 0.137 ng/mL (1.991–2.536, 95% CI) in control men, 2.120 ± 0.175 ng/mL (1.774–2.466, 95% CI) in control women, 3.258 ± 0.147 ng/mL (2.966–3.550, 95% CI) in cocaine-addicted men and 3.180 ± 0.259 ng/mL (2.666–3.693, 95% CI) in cocaine-addicted women. Multiple comparisons of these adjusted means indicated that the cocaine group had significantly higher plasma OEA concentrations than did the controls in both men (**p < 0.01) and women (***p < 0.001).

Two polyunsaturated *N*-acyl-ethanolamines, AEA and LEA, were also measured and statistically analyzed. Similar to OEA and the saturated lipid mediators, history of cocaine addiction produced a main effect on plasma AEA concentrations (*F*_1,122_ = 38.59, *p* < 0.001) (Figure 3E). However, there was a significant effect of BMI (*F*_1,122_ = 6.29, *p* = 0.014) that explained 5.8% of the variance. The estimated marginal means (age = 39.41 and BMI = 25.34) of AEA concentrations were as follows: 0.467 ± 0.032 ng/mL (0.404–0.530, 95% CI) in control men, 0.421 ± 0.040 ng/mL (0.341–0.501, 95% CI) in control women, 0.668 ± 0.034 ng/mL (0.601–0.736, 95% CI) in cocaine-addicted men and 0.753 ± 0.060 ng/mL (0.634–0.872, 95% CI) in cocaine-addicted women. The paired comparisons showed that both cocaine-addicted men and women had a significant increase in AEA concentrations (**p < 0.01 and ***p < 0.001, respectively) compared with their respective controls.

When LEA concentrations were analyzed (Figure 3F), we observed a significant primary effect of history of cocaine addiction (*F*_1,122_ = 45.80, *p* < 0.001). Here, an interaction between both factors was also detected (*F*_1,122_ = 5.58, *p* = 0.020). BMI and age had no effect on LEA concentrations. Multiple comparisons showed significant increases in the plasma LEA concentrations of cocaine-addicted men (***p < 0.001) and women (***p < 0.001) in comparison with their respective controls, but we observed no differences between both sexes.

Overall, free *N*-acyl-ethanolamine concentrations were found to be increased in the plasma of cocaine-addicted subjects with no effect of sex. However, there were two exceptions: (i) SEA concentrations were decreased in the cocaine group and (ii) POEA concentrations were increased exclusively in cocaine-addicted women. The covariates age and BMI were found to be significantly related to the plasma concentrations of certain acyl derivatives (i.e., POEA, OEA, and AEA).

#### 2-acyl-glycerols

As shown in Figure 4, the plasma concentrations of 2-AG and 2-LG were determined to be glycerol-derived molecules.

Statistical analysis revealed a significant main effect of history of cocaine addiction (*F*_1,122_ = 7.60, *p* = 0.007) on 2-AG concentrations (Figure 4A). There was a significant relationship between age and 2-AG concentrations (*F*_1,122_ = 9.85, *p* = 0.002), and the covariate explained 8.7% of the variance. The adjusted means (age = 39.41 and BMI = 25.34) of 2-AG concentrations and standard errors were as follows: 6.869 ± 0.508 ng/mL (5.861–7.877, 95% CI) in control men, 5.779 ± 0.646 ng/mL (4.497–7.060, 95% CI) in control women, 5.116 ± 0.545 ng/mL (4.035–6.197, 95% CI) in cocaine-addicted men, and 3.749 ± 0.958 ng/mL (1.849–5.650, 95% CI) in cocaine-addicted women. Paired comparisons did not indicate significant differences among groups.

Finally, we also found a significant primary effect of history of cocaine addiction (*F*_1,122_ = 16.57, *p* < 0.001) on plasma 2-LG concentrations. We observed no effects of age or BMI on the 2-LG concentrations. The *post hoc* tests indicated significant decreases in the 2-LG concentrations of cocaine-addicted subjects compared with the control group for both men (***p < 0.001) and women (*p < 0.05).

Unlike *N*-acyl-ethanolamines, the plasma concentrations of glycerol derivatives were decreased in the cocaine group, but sex differences were not found. Interestingly, there was a robust relationship between BMI and 2-AG concentrations.

### Plasma concentrations of chemokines, cytokines, and fatty acid derivatives in subjects with a history of cocaine addiction

The plasma concentrations of chemokines, cytokines, and fatty acid derivatives in the cocaine group were analyzed by univariate general linear models to evaluate their relationships with cocaine symptom severity, diagnosis of comorbid psychiatric disorders, and length of cocaine abstinence (Table 4). Additionally, the relationships with BMI, age, and sex were also evaluated.

**Table 4:** **Multiple relationships between plasma concentrations of chemokines, cytokines, and fatty acid derivatives (dependent variables) and independent variables in abstinent cocaine-addicted subjects**.

Dependent variable	Main effect (independent variable)^a^											
	Sex		Body mass index		Age		Length of cocaine abstinence		Cocaine symptom severity		Comorbid psychiatric disorders	
	*F*	*p*-value	*F*	*p*-value	*F*	*p*-value	*F*	*p*-value	*F*	*p*-value	*F*	*p*-value
**UNIVARIATE GENERAL LINEAR MODELS**												
CX3CL1 (fractalkine)	2.194	ns	0.930	ns	0.222	ns	0.010	ns	2.469	ns	0.354	ns
CCL2 (MCP-1)	0.005	ns	0.949	ns	0.657	ns	0.524	ns	0.687	ns	3.204	ns
CXCL12 (SDF-1)	0.452	ns	0.018	ns	0.026	ns	0.001	ns	2.651	ns	0.011	ns
IL-1β	1.162	ns	0.433	ns	0.545	ns	0.121	ns	2.550	ns	2.434	ns
IL-6	0.012	ns	0.272	ns	0.854	ns	0.411	ns	1.872	ns	3.690	ns
IL-10	2.103	ns	1.091	ns	2.407	ns	1.791	ns	1.033	ns	2.765	ns
TNFα	0.165	ns	0.004	ns	0.036	ns	0.076	ns	0.218	ns	3.032	ns
SEA	0.004	ns	0.774	ns	0.266	ns	1.408	ns	1.842	ns	**11.49**	**0.002**
PEA	1.246	ns	0.911	ns	1.116	ns	0.490	ns	1.746	ns	1.858	ns
OEA	0.300	ns	0.095	ns	1.181	ns	0.098	ns	0.501	ns	2.177	ns
POEA	**12.88**	**0.001**	**6.798**	**0.013**	0.011	ns	0.427	ns	0.185	ns	0.037	ns
AEA	0.453	ns	**4.578**	**0.039**	0.166	ns	0.265	ns	2.842	ns	2.691	ns
LEA	2.601	ns	3.458	ns	0.144	ns	0.138	ns	1.584	ns	0.946	ns
2-AG	**4.593**	**0.039**	0.149	ns	**6.619**	**0.014**	0.503	ns	0.004	ns	2.288	ns
2-LG	**6.703**	**0.014**	0.063	ns	2.981	ns	0.003	ns	0.692	ns	**6.897**	**0.012**

*^a^Tests of between-subjects effects*.

*ns, non-significant*.

*Bold indicates statistically significant *p*-values*.

We observed no relationships between plasma concentrations of chemokines and cytokines and such variables. In fact, the main effect of sex on cytokine concentrations in the control and cocaine groups was not observed when only the cocaine group was analyzed. However, the plasma concentrations of several fatty acid derivatives were found to be associated with certain variables.

Thus, SEA concentrations were related to the diagnosis of comorbid psychopathologies (*p* < 0.01), and an increase in SEA concentrations was observed in cocaine-addicted subjects diagnosed with psychiatric comorbidity. As previously observed, POEA and AEA concentrations in the cocaine group were found to be affected by BMI (*p* < 0.05). Additionally, there was a main effect of sex (*p* = 0.001) on POEA concentrations. Regarding 2-acyl glycerol derivatives, whereas 2-AG concentrations were significantly affected by age and sex (*p* < 0.05), 2-LG concentrations were affected by diagnosis of psychiatric comorbidity and sex (*p* < 0.05). Similar to SEA, an increase in 2-LG concentrations was observed in cocaine subjects diagnosed with psychiatric comorbidity.

Thus, among the independent variables related to cocaine addiction in the cocaine group that were evaluated, we only found significant associations between comorbid psychiatric disorders and fatty acid derivative concentrations (i.e., SEA and 2-LG).

## Discussion

The present findings show that sex is a relevant modulatory factor in the presence of comorbid mental and substance use disorders in individuals with lifetime cocaine use disorders. As expected, cocaine-addicted men seeking treatment were more common than women. Whereas the abstinent cocaine-addicted men were characterized by increased rates of other substance use disorders such as alcohol and marijuana, the cocaine-addicted women showed a higher prevalence of comorbid mental disorders, such as mood, anxiety, and psychotic disorders. Additionally, the plasma concentrations of putative biomarkers for cocaine addiction and comorbidity were influenced by sex. In the present study, we examined sex differences in inflammatory mediators and fatty acid derivatives because these molecules were reported to be affected by the pathological use of cocaine (19, 20). The most relevant findings were that all of the evaluated inflammatory cytokines and the monounsaturated fatty acid derivative POEA were found to be differentially altered in cocaine-addicted women, extending the influence of sex to plasma biomarkers for cocaine addiction. In addition, SEA and 2-LG concentrations were associated with psychiatric comorbidity in abstinent cocaine-addicted subjects.

In the present study, subjects diagnosed with lifetime cocaine use disorders showed high rates of mental and other substance use disorders, as reported previously in cocaine users and abstinent cocaine-addicted subjects (11, 12, 27), and there were differences in the psychiatric prevalence according to sex. Several epidemiological studies have observed significant sex differences among subjects diagnosed with mood and anxiety disorders. The incidence of mental disorders is enhanced in women compared with men, particularly in periods of life such as pregnancy, maternity, menopause, or after traumatic events (15). These elevated rates of lifetime psychiatric disorders in women have also been reported with substance use disorders (14, 28). Consistent with these previous studies, we diagnosed a higher prevalence of comorbid mental disorders (i.e., mood, anxiety, and psychosis) in women, whereas higher rates of other substance use disorders (especially with alcohol) were observed in cocaine-addicted men. As expected, the lifetime cocaine symptom severity was severe in both sexes with no differences with respect to length of abstinence and duration of cumulative cocaine use.

Collectively, these findings suggest that any potential biomarker for cocaine addiction and psychiatric comorbidity must account for the influence of sex. Recent evidence indicates that cocaine and other psychostimulants alter the peripheral concentration of circulating mediators that may influence the cognitive and behavioral changes associated with the process of addiction. Among these mediators, we assessed the plasma concentrations of inflammatory mediators and fatty acid derivatives. Chemokines and proinflammatory mediators are affected by cocaine symptom severity, whereas anti-inflammatory fatty acid derivatives such as endocannabinoids and their congeners are affected by the history of pathological use of cocaine and the presence of comorbid disorders. However, the impact of sex has not been sufficiently studied because the female population seeking outpatient treatment in centers for cocaine addiction is very low, as previously indicated (1).

### Chemokine and cytokine concentrations

Growing evidence has demonstrated naturally occurring sex differences in the immune response and inflammatory mediators. During their reproductive years, women have a more potentiated cellular and humoral immune response than do men (29). Indeed, it is thought that fluctuations in estrogen may alter immune cell function by affecting cytokine and chemokine production (30). Furthermore, several recent reports have related inflammatory mediators in blood and cerebrospinal fluid to addiction to drugs of abuse, such as alcohol, psychostimulants, and opiates (31).

Chemokines are chemoattractants that are involved in leukocyte trafficking to the site of inflammation (32), but they also play an important role in neuronal development, maturation, survival, and regeneration in the central nervous system (CNS) (33). Therefore, cerebral changes underlying addiction and psychiatric comorbidities might be reflected in peripheral alterations. Recently, chemokines have been proposed as pathologically relevant biomarkers or therapeutic targets for psychiatric disorders (34). In fact, we found that the CCL2 and CXCL12 concentrations were decreased in both male and female cocaine users. These changes in circulating chemokines are in accordance with the results from another study in abstinent cocaine-addicted population, which suggested that these chemokines were biomarkers for the pathological use of cocaine, although we observed no changes in plasma CX3CL12 concentrations (20). Moreover, we detected no differences by sex in the plasma concentrations of these chemokines.

In addition to chemokines, we evaluated plasma cytokine concentrations, including pro- and anti-inflammatory mediators. In this case, we found a clear sexual dimorphism in the circulating concentrations of all of the cytokines assessed in the present study. The plasma concentrations of IL-1β, IL-6, IL-10, and TNFα were higher in healthy women compared with men. Interestingly, these differences were absent in subjects diagnosed with lifetime cocaine use disorders.

Previous studies have reported that chronic exposure to drugs of abuse, such as alcohol and cocaine, suppresses immune responses. Contradictory data have been published in relation to alcoholism. For instance, whereas a significant increase in the production of IL-1β, IL-6, IL-12, and TNFα was observed in chronic alcoholics without liver disease, chronic alcoholics with liver disease who were drinking alcohol showed low production of IL-1β and TNFα (35). Regarding cocaine, it has been reported that cocaine-dependent subjects show a decreased capacity to express proinflammatory cytokines such as TNFα and IL-6 in monocytes (36). Further, we have recently shown that abstinent cocaine-addicted subjects have low TNFα concentrations and IL-1β concentrations, and that they are affected by the cocaine symptom severity (20). Our data may be related to long-term cocaine-induced changes in cytokine concentrations, but these changes were only produced in women with no effect in male addicts. The plasma concentrations of inflammatory cytokines were clearly reduced in women, and such cytokine reduction might be associated with decreased immune activity and an increased risk of developing mental disorders (15, 37). However, plasma concentrations of chemokines and cytokines were not associated with sex and comorbid psychopathologies in cocaine-addicted subjects.

### Fatty acid derivative concentrations

Several lines of evidence suggest that bioactive lipids, such as endocannabinoids and related congeners, are involved in the acquisition and maintenance of drug-taking behavior and other processes associated with addiction (38, 39). However, all of these studies are based on preclinical observations because there are few studies concerning plasma lipid derivatives in individuals with substance use disorders (40, 41), principally with alcohol use (42, 43). Endocannabinoids act on cannabinoid receptors to exert their effects and are composed of two structural types of lipid derivatives: *N*-acyl-ethanolamines (e.g., AEA) and 2-acyl-glycerols (e.g., 2-AG).

Clinical studies in alcohol users reported that alcohol use affects circulating endocannabinoid concentrations after moderate and chronic consumption (42, 43). Regarding cocaine use, we recently showed that *N*-acyl-ethanolamine concentrations are increased in abstinent cocaine addicts, whereas both 2-AG and 2-LG are reduced (19). Interestingly, distinct profiles of both endocannabinoid types (AEA and 2-AG) have also been described in distinct brain areas after administering drugs of abuse in rodents (44, 45). In agreement with these studies, the present study shows that plasma concentrations of all lipid-derived molecules were distinctly affected by history of cocaine addiction. All *N*-acyl-ethanolamines, except SEA, were found to be increased in cocaine-addicted subjects, but 2-acyl-glycerols were decreased in relation to healthy subjects. Overall, we observed no sex differences in the changes of plasma concentrations of fatty acid derivatives. However, the POEA concentrations were found to be altered exclusively in women diagnosed with lifetime cocaine use disorders. Our current data show that plasma POEA was markedly increased in abstinent cocaine-addicted women, and similar changes have been previously observed in another study with cocaine addicts who were diagnosed with comorbid mood or anxiety disorders (19). Little is known about POEA, but it has been suggested that it regulates appetite and energy metabolism through a non-cannabinoid receptor (46, 47). Interestingly, palmitoleate is the fatty acid from which POEA is derived, and the former has been suggested to be a lipokine associated with metabolic abnormalities (48). In fact, we observed a significant effect of BMI on POEA concentrations of our sample, and this influence stayed with the cocaine group. However, there are no data related to the role of POEA in psychopathologies and addiction. In the present study, we did not observe relationships between POEA concentrations and variables related cocaine addiction such as length of abstinence, cocaine symptom severity, or diagnosis of comorbid psychopathologies, but sex was strongly associated with POEA in cocaine-addicted subjects. Although the small number of cocaine-addicted women does not allow for a conclusion of the impact of comorbid mental disorders on POEA concentrations, we must note that female addicts have a higher prevalence of psychiatric comorbidity than do men.

The associations between circulating endocannabinoids and mental disorders have been extensively studied in clinical studies, and a common observation found in these disorders is the elevated concentrations of *N*-acyl-ethanolamines (49–51). Further, increased *N*-acyl-ethanolamine concentrations have been observed in psychiatric patients with substance use disorders, such as in schizophrenia patients (40). Accordingly, we observed a significant relationship between SEA and diagnosis of comorbid psychiatric disorders in cocaine-addicted subjects, with higher concentrations in comorbid addicts. However, classical endocannabinoids such as AEA and 2-AG were not influenced by comorbidity.

### Limitations and future perspectives

Our findings support the importance of monitoring these putative predictors in the context of a history of cocaine addiction by accounting for both sex differences and psychiatric comorbidity.

We are aware of the limitations of the current study. First, the number of cocaine-addicted women is small, and replicating these data with a larger sample will be necessary to confirm the sexual dimorphism observed in the present study, primarily in plasma cytokines and POEA. Second, we must determine whether these alterations in cocaine-addicted women are exclusively attributable to sex or to comorbid psychiatric disorders. Therefore, new studies in female psychiatric patients with no history of drug abuse will be necessary to elucidate the influence of mental disorders in these circulating predictors of cocaine addiction. Related to these mentioned limitations, women were randomly recruited without considering the menstrual cycle, and this condition should be considered in future studies including cocaine-addicted women. Third, with regard to the cocaine use effect, we are unaware of the effects on the plasma concentrations of these molecules in active cocaine users because the presence of cocaine can induce prolonged activation, and the present data were obtained from subjects with a history of addiction. Animal models can be a useful tool to perform future investigations for responding to these questions. Finally, the recruitment of abstinent cocaine-addicted subjects under treatments in centers for addiction was performed on an outpatient basis; consequently, medications as well as social and environmental factors must be considered.

In summary, whereas the plasma concentrations of inflammatory and fatty acid-derived mediators may allow for a better stratification of cocaine addicts, the sexual dimorphism must also be considered for the adequate selection of biomarkers for cocaine addiction and therapeutic purposes.

## Author Contributions

FRF and FJP were responsible for the study concept and design. MP, PA, NGM, and JJR coordinated and recruited participants from outpatient treatment centers for addiction. MP, PA, NGM, and PR-S contributed to the acquisition of psychiatric data by means of interviews. AS, JS, and ECO processed the blood samples. AP and RdT supervised and performed the benzoylecgonine detection and the quantification of fatty acid derivatives in plasma. VB, JAC, and JA supervised and performed the quantification of cytokines and chemokines in plasma. FJP, AS, and PA assisted with data analysis and interpretation of findings. FRF and FJP drafted the manuscript. MT, RdT, and JA provided critical revision of the manuscript for important intellectual content. All the authors critically reviewed the content and approved the version for publication.

## Conflict of Interest Statement

The authors declare that the research was conducted in the absence of any commercial or financial relationships that could be construed as a potential conflict of interest.

## Abbreviations

2-AG, 2-arachidonoyl-glycerol; 2-LG, 2-linoleoyl-glycerol; AEA, *N-*arachidonoyl-ethanolamine; CCL2/MCP-1, chemokine (C-C motif) ligand 2/monocyte chemotactic protein-1; CXCL12/SDF-1, chemokine (C-X-C motif) ligand 12/stromal cell-derived factor-1; CX3CL1/fractalkine, chemokine (C-X_3_-C motif) ligand 1/fractalkine; IL-1β, interleukin-1 beta; IL-6, interleukin-6; IL-10, interleukin-10; LEA, *N*-linoleoyl-ethanolamine; OEA, *N*-oleoyl-ethanolamine; PEA, *N*-palmitoyl-ethanolamine; POEA, *N*-palmitoleoyl-ethanolamine; SEA, *N*-stearoyl-ethanolamine; TNFα, tumor necrosis factor-alpha.
